# The Iterative Design and Development of an Affordable Ultrasound Simulator

**DOI:** 10.7759/cureus.52300

**Published:** 2024-01-15

**Authors:** Anjali Jagannathan, Julia Micallef, Tim Clarke, Kristin Armstrong, Adam Dubrowski

**Affiliations:** 1 Human Kinetics, University of Ottawa, Ottawa, CAN; 2 Health Sciences, Ontario Tech University, Oshawa, CAN; 3 Engineering and Applied Sciences, Ontario Tech University, Oshawa, CAN; 4 Clinical Applications, FUJIFILM Sonosite Canada Inc, Toronto, CAN

**Keywords:** point-of-care ultrasound, train-the-trainer, ultrasound phantom, additive manufacturing, simulation-based learning, simulation-based medical education, three-dimensional (3d) printing

## Abstract

Simulation-based medical education (SBME) offers a secure and controlled environment for training in ultrasound-related clinical skills such as nerve blocking and intravenous cannulation. Sonographer training for point-of-care ultrasound often adopts the train-the-trainer (TTT) model, wherein a select group of sonographers receive on-site training to subsequently instruct others. This model traditionally relies on expensive commercial ultrasound simulators, which presents a barrier to the scale-up of the TTT model.

This study aims to address the need for cost-effective ultrasound simulators suitable for both initial and cascaded TTT. The objective of this report is to present the design and development of an affordable ultrasound simulator, which mimics anatomical features under ultrasound. The simulator was created using additive manufacturing techniques, including 3D printing, ballistic gel, and silicone work.

We report on three development-feedback iterations, with feedback provided by an experienced sonographer from FUJIFILM Sonosite Canada Inc. using the think-aloud approach. Overall the results indicate that de-gassed silicone may serve as a good medium; vessels are best produced as hollow canals within the de-gassed silicone; 3D-printed bones cast acoustic shadows, which are reduced by increasing rigidity of the structures, and 3D printing filament and silicone can be used for nerve bundles. Future developments will focus on achieving anatomical accuracy, exploring alternative materials and printing parameters for the bones, and analyzing embedded structures at varying depths within the silicone. The next steps involve integrating the simulator into ultrasound curricula for a formal assessment of its effectiveness as a training tool.

## Introduction

Obtaining proficiency in ultrasound skills is a crucial element in numerous healthcare education programs [1]. Simulation-based medical education (SBME) offers trainees a secure and controlled environment to acquire, enhance, and sustain their ultrasound-related proficiencies, such as nerve blocking and intravenous cannulation [2].

In the last ten years, there has been a noteworthy surge in research and development efforts aimed at crafting "do-it-yourself" (DIY) ultrasound simulators utilizing readily available materials [3]. Notably, innovations in gelatin-based simulators have shown promise, with the primary ultrasound-compatible substance being ballistic gel (composed of water, gelatin, and metamucil). Other materials like tofu, raw meat, and spam have also displayed some degree of effectiveness [4]. However, these "DIY" simulators lack uniformity and cannot be systematically employed for training and assessment, while animal-based simulators present logistical and ethical challenges.

Procuring a commercially available simulator is a viable but costly solution. For instance, the "gold standard" in ultrasound simulation training is the CAE Blue Phantom Ultrasound Simulator. This simulator offers advanced educational features, precise imaging, and an authentic learning experience. However, the CAE Blue Phantom is among the most expensive ultrasound simulators in the market, with the basic vascular ultrasound training block priced at $812.38 Canadian dollars (CAD) per unit [5].

Through the utilization of additive manufacturing techniques, such as 3D printing and silicone modeling, it may be feasible to create cost-efficient, anatomically accurate, and standardized simulators tailored for ultrasound training [6]. When appropriately degassed, addition-cure silicone has demonstrated suitability for ultrasound training, though it is limited by the depth and image distortion stemming from elements embedded within the silicone (e.g., bones, nerves, and vessels). 3D-printed bones manufactured from polylactic acid (PLA) filaments have also proven effective in delivering satisfactory image quality within ultrasound-compatible simulators. However, image quality is contingent on the 3D printing parameters and materials employed [7]. Given recent strides in 3D printing and material sciences, it is imperative to reconsider the role of additive manufacturing in crafting standardized simulators encompassing anatomical structures such as bones, vessels, and nerves incorporated within degassed silicone.

Hence, the primary focus of this technical report is to elucidate the process of devising a cost-effective and anatomically precise ultrasound simulator using 3D printing and silicone modeling.

## Technical report

Context

The simulator outlined in this technical report was developed and tested as part of a collaborative effort involving industry partner FUJIFILM Sonosite Canada Inc., a point-of-care ultrasound machine company. It was specifically designed to align with the train-the-trainer (TTT) training model, a strategy that empowers a wide spectrum of healthcare trainees to access customized and budget-friendly learning experiences [8]. In contrast to directly training end-users, the TTT model places a significant emphasis on equipping trainers with the requisite skills and knowledge to deliver tailor-made training sessions suited to each institution's unique needs. This approach has demonstrated its effectiveness in enhancing the acquisition of ultrasound skills and significantly boosting learners' expertise in this field [8]. For instance, Butki et al. reported that using the TTT model resulted in an 87.5% increase in knowledge among emergency medicine residents, as assessed through pre- and post-training evaluations [8].

FUJIFILM Sonosite Canada Inc., as our industrial partner, routinely employs the TTT approach. In this approach, a clinical applications specialist provides training to a group of trainers at a single location or institution (such as a hospital). Subsequently, these trained trainers are tasked with disseminating their acquired knowledge throughout the institution. While FUJIFILM Sonosite supports the development of skills and knowledge within this TTT model, the trainers currently need to procure or create their own simulators to deliver TTT within their clinical and educational settings. Addressing this need for a cost-effective simulator, which can be integrated into a more comprehensive training package within the TTT model, has been identified by our partner as a valuable enhancement to the existing training approach.

Inputs

Personnel

The simulator (referred to as the maxSIMghost) was co-designed by two groups, which we will refer to as the research and the clinical teams. The research team consisted of three researchers (AJ, JM, AD) and a 3D designer (TC) from maxSIMhealth laboratory (maxSIMhealth.com), a research laboratory located at Ontario Tech University in Oshawa, Ontario, Canada. The clinical team consisted of a clinical applications specialist representative from FUJIFILM Sonosite Canada Inc., who has been practicing ultrasound sonography for seventeen years and worked in ultrasound training and product demonstration for six years (KA), as well as a staff anesthesiologist from the Thunder Bay Regional Health Sciences Centre, Thunder Bay, Ontario, Canada. 

Equipment and Materials

Table 1 shows the equipment and materials used to manufacture the simulator. 

**Table 1:** Equipment and materials used to produce the simulator. PLA, polylactic acid

	Equipment and materials
1	Ultimaker S5 3D printer (Ultimaker B.V., Utrecht, The Netherlands)
2	Ultimaker Cura 3D printing software (Ultimaker B.V., Utrecht, The Netherlands)
3	Fusion360™ (Autodesk Inc., San Rafael, CA, USA)
4	TinkerCAD™(Autodesk Inc., San Rafael, CA, USA)
5	SolidWorks 2022
6	Ecotough™ PLA filament material (Mississauga, ON)
7	Ecoflex™ 00-20 FAST silicone (Smooth-On, Macungie, PA, USA)
8	Ecoflex™ 00-35 FAST silicone (Smooth-On, Macungie, PA, USA)
9	Silc-Pig™ coloring (Smooth-On, Macungie, PA, USA)
10	Ballistic Gel (16 oz water, 500 mL glycerin, 16 packs Knox gelatin, acrylic paint)
11	Rotary Vane Vacuum Pump (FJC (6909) 3.0 CFM Vacuum Pump)
12	1L Plastic Cup
13	Metal Rods (GLARKS, Shenzhen, China)
14	Ease Release 200 (Sculpture Supply Canada, Toronto, ON)
15	Yesallwas silicone rubber tubing
16	Elegoo Standard Grey Resin (405 nm)

Process

The development and testing process encompassed three rounds. Initially, the clinical team established the following criteria: 1) The design must be cost-effective, with the maximum cost per simulator not exceeding $20 CAD, as this price point was deemed acceptable by the company to be considered as consumable costs of on-site training. 2) The design should incorporate bones, vessels, and nerves, as the clinical team was ultimately interested in designing a simulator for ultrasound-guided nerve blocks in the knee (popliteal fossa) and the elbow (cubital fossa), which encompass these three anatomical structures. 

Each round involved the research team creating a set of test simulators. During testing, a clinical applications specialist performed ultrasound scans on these simulators while being kept unaware of specific design details, materials used, and construction. To maintain the blinding, identical simulators were produced and labeled alphabetically. The clinical applications specialist utilized the SonoSite Edge II ultrasound system and followed the think-aloud observation (TAO) protocol, which involves verbalizing thoughts during tasks to reveal cognitive processes [9,10]. This approach is commonly employed in research, usability testing, and education to gain insights into decision-making, problem-solving strategies, and product design issues [10]. The research team took field notes for subsequent simulator refinement.

Round 1

The primary objective of the first round was to assess the feasibility of using ballistic gel and silicone as media for housing the anatomical structures within the simulator. A secondary objective was to assess the degassing process for optimizing ultrasound imaging.

Methods: To achieve these objectives, the research team produced five distinct prototypes, three using silicone (n=3) and two using ballistic gel (n=2) (see Table 2).

**Table 2:** Prototypes A to E created in Round 1. For each prototype, this table demonstrates the materials used (column 2), as well as the degassing stages and processes (column 3).

Prototype	Material	Degassing
A	Silicone	Stage 1: Degassed in a plastic cup, then poured into the 3D-printed mold
B	Silicone	Stages 1 and 2: Degassed in a plastic cup, then again in the 3D-printed mold
C	Silicone	Stage 2: Degassed only in the 3D-printed mold
D	Ballistic gel	Stage 1: Degassed in a plastic cup, then poured into the 3D-printed mold
E	Ballistic gel	No degassing

**Figure 1:**
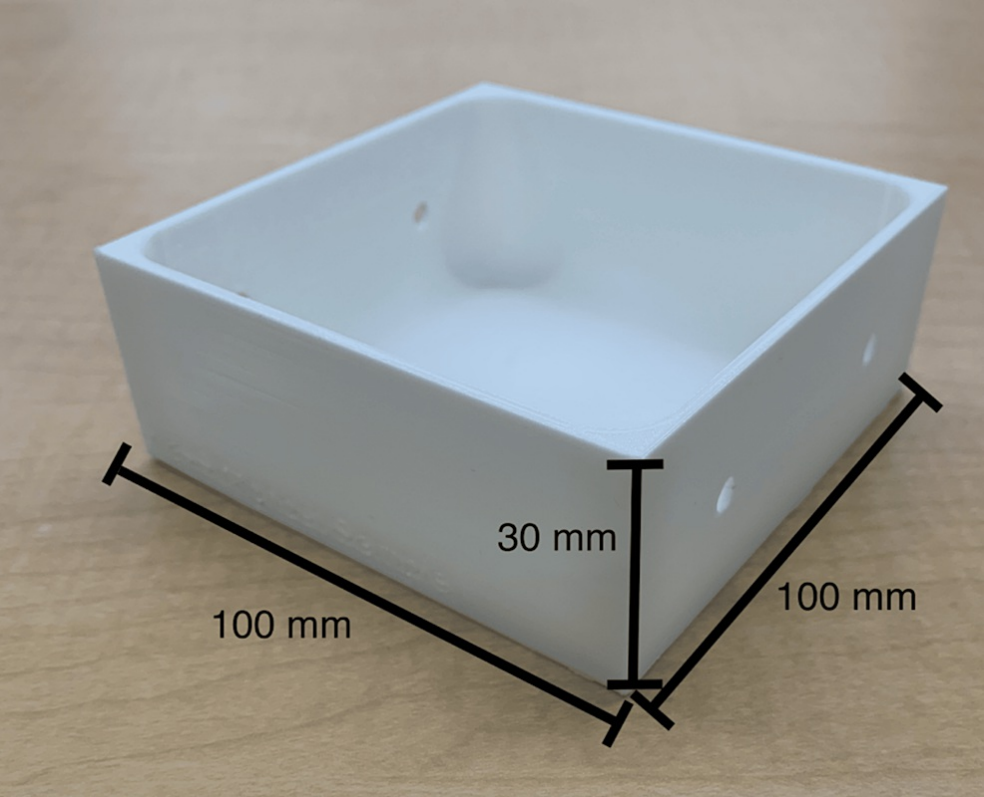
3D-printed mold used in Round 1. The mold was designed to construct prototypes A to E and was 3D-printed using Ecotough™ PLA filament material on an Ultimaker S5 3D printer. Two round openings were incorporated in the design on two opposite sides of the mold to allow for the insertion of anatomical structures such as veins and nerve bundles during silicone pouring. PLA, polylactic acid

In the first step, a 3D-printed mold for the phantom (Figure 1) was digitally designed using Fusion360™ (Autodesk Inc., San Rafael, CA, USA), 3D sliced with Ultimaker Cura 3D printing software, and 3D-printed using Ecotough™ PLA filament material (Mississauga, ON) on an Ultimaker S5 3D printer (Ultimaker B.V., Utrecht, The Netherlands).

This resulted in five different simulator prototypes (Table 2). Prototypes A, B, and C were produced using silicone, while prototypes D and E were fashioned with ballistic gel. Silicone prototypes used Ecoflex™ 00-20 FAST silicone (Smooth-On, Macungie, PA, USA) and Silc-Pig™ coloring (Smooth-On, Macungie, PA, USA). Ballistic gel prototypes consisted of 16 oz of water, 500 mL of glycerin, 16 packs of Knox gelatin, and acrylic paint.

To optimize ultrasound penetration, some prototypes (A-D) underwent degassing, while E was not degassed. Previous research indicated that degassing silicone was crucial for this purpose; however, there is no mention in the literature about how degassing affects ballistic gel. The Rotary Vane Vacuum Pump (FJC (6909) 3.0 CFM Vacuum Pump) was used to degas the silicone. This process involved placing the uncured silicone in a vacuum chamber, increasing the pressure to 2500 kpa until bubbles expanded, and then reducing the pressure to 0 kpa once the bubbles cleared from the surface [6].

For the silicone prototypes (A-C) three degassing stages were tested: (1) pre-pouring, (2) pre- and post-pouring, and (3) post-pouring. In the pre-pouring stage, a 1 L plastic measuring cup was used to mix the two-part Ecoflex™ 00-20 FAST silicone solution with Silc-Pig™ coloring for a total volume of 300 mL of silicone. For prototypes A and B, the cup was immediately placed in the degassing chamber. After degassing, the silicone was transferred to the mold for prototype A and to the mold in the case of prototype B for the second stage of degassing before setting to cure. Prototype C differed in that the silicone was poured directly into the mold and subjected to only the second degassing stage. Subsequently, it was allowed to cure.

For the ballistic gel prototype (D), a single degassing stage was implemented. This involved placing the gel in a measuring cup and following the same degassing procedure before pouring into the mold and setting to cure. Prototype E, on the other hand, was poured directly into the mold without any degassing before setting to cure.

The primary comparisons of interest were prototypes A and B to assess the impact of the second stage of degassing on ultrasound image quality. Prototypes A and C to evaluate the effects of initial degassing in a measuring cup versus the 3D-printed mold. Prototypes D and E to test the impact of degassing on ballistic gel. Subsequently, a comparison between A and E was made to examine the difference in ultrasound image quality between silicone and ballistic gel.

**Figure 2:**
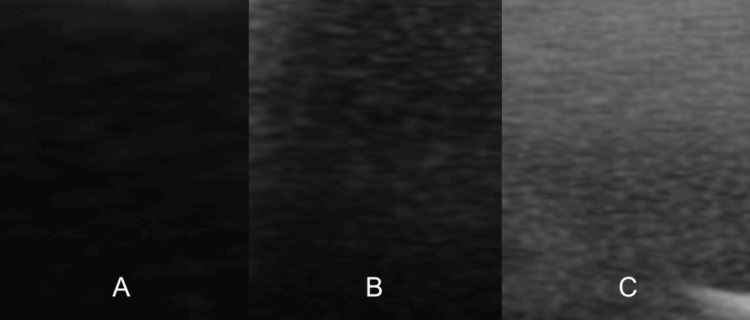
Image quality of prototypes A (left), B (middle), and C (right). Images B and C demonstrate the presence of air bubbles trapped in prototypes B and C, which reduces the ultrasound properties of the simulator.

Results: The results of testing prototypes A and B revealed that the second stage of silicone degassing (pre- and post-pouring) did not improve the image quality (Figure 2). On the contrary, the second degassing of silicone showed the presence of air bubbles, as indicated in Figure 2. One plausible explanation for this is that the 3D-printed materials used to manufacture the mold contained air particles, even if the infill parameters were set to 100%. This was confirmed by the comparison of the quality of images generated from comparisons of prototypes A and C, which showed that when the initial degassing was done in the 3D-printed mold, the air particles were also present (Figure 2).

**Figure 3:**
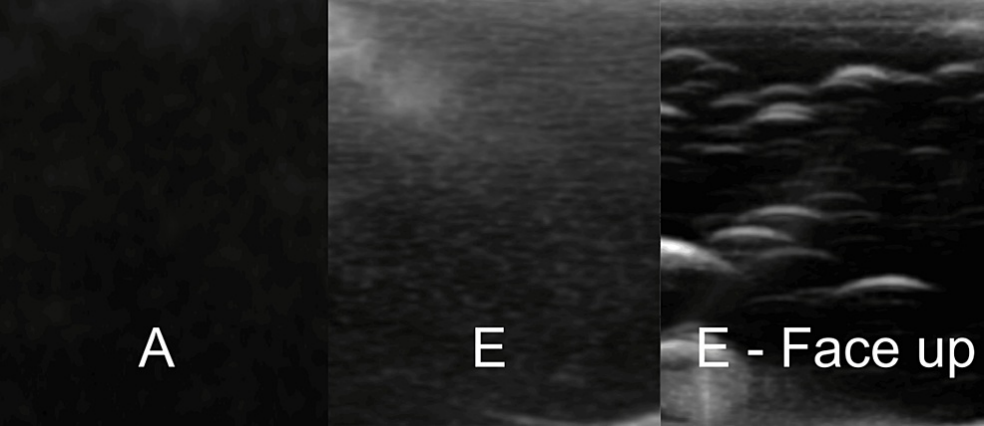
Image quality of prototype A on the left (optimal silicone), E in the middle (optimal ballistic gel), and the surface of prototype E that faced up during the curing process on the right. Image E demonstrates the presence of air bubbles trapped in prototype E, which reduce the ultrasound properties of the simulator. Image E - Face up demonstrates the increased amount of air bubbles trapped close to the surface, further reducing the ultrasound properties of the simulator.

The ballistic gel prototypes were compared, and there were significantly less air bubbles without the degassing process, greatly improving the image quality. This was especially pronounced when the prototypes were imaged over the surface that faced up during the curing process (Figure 3). This indicates that the degassing process led to partial degassing and the air bubbles were trapped near the surface of the prototype. Finally, the quality of images between the optimal silicone and ballistic gel prototypes (i.e., A and E) showed an advantage for the first stage (pre-poured) degassed silicone-based prototype, although the gain on the ultrasound machine needed to be set higher to obtain clear images (Figure 3).

Round 2

The primary aim of the second round was to assess how different manufacturing materials, 3D printing filaments, and parameters influenced the quality of images when nerves and vessels were integrated into the silicone prototypes.

Methods: To execute this, three distinct types of vessels and two types of nerves were fabricated and incorporated into the simulators. The mold from Round 1 was redesigned to feature three sets of parallel holes, each with a 6 mm diameter, to accommodate the nerves and two vessels in each prototype. The prototypes underwent degassing using the same first-stage degassing protocol (pre-pouring) employed with prototype A in Round 1.

Vessels: Three different types of vessels were tested. 

Vessel type 1: Silicone rubber tubing with dimensions 3 mm internally and 5 mm externally (Yesallwas silicone rubber tubing) was used, acquired through amazon.ca [11]. The tubing was cut into two segments, each 20 cm long. Before pouring the degassed silicone, these segments were threaded through pairs of parallel holes in the 3D-printed mold. Syringes were employed to fill the vessels with water prior to testing.

**Figure 4:**
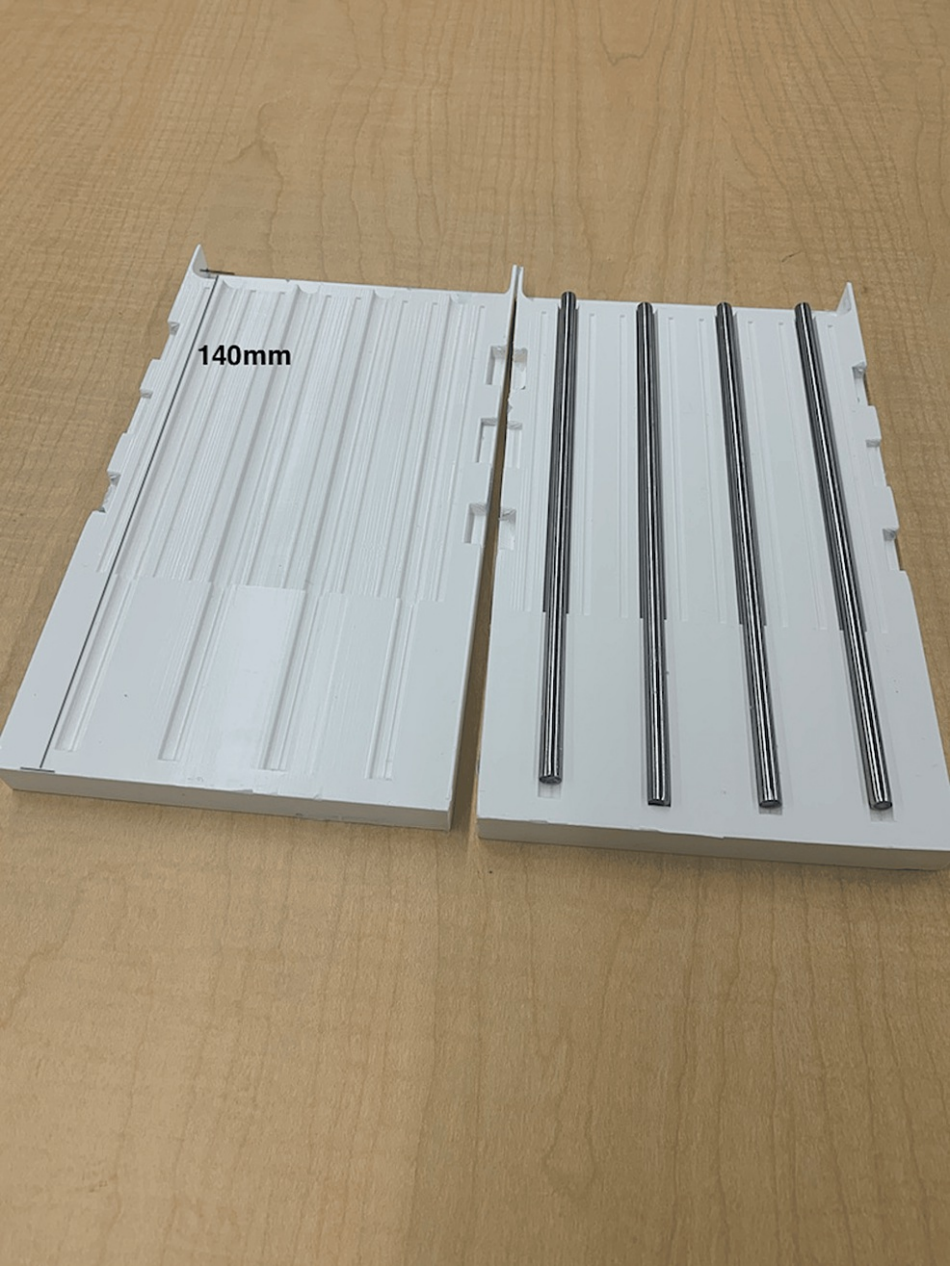
A custom-developed 3D-printed mold and commercially purchased metal rods used to develop vessel type 2. The mold was 3D-printed using Ecotough™ PLA filament material on an Ultimaker S5 3D printer. The four channels were designed to create silicone tubes each measuring 140 mm in length, with diameters ranging from 7 mm to 10 mm. To create the openings, metal rods with a 6 mm diameter were inserted into the channels of the mold before pouring the silicone to create tubes with a wall thickness of 1-1.5 mm. PLA, polylactic acid

Vessel type 2: This vessel was fashioned using a procedure from a previous report for a bile duct anastomosis simulator [12]. The mold was designed in Fusion360™, 3D sliced with Ultimaker Cura 3D printing software (Ultimaker B.V., Utrecht, The Netherlands), and 3D-printed using Ecotough™ PLA filament material on an Ultimaker S5 3D printer. The mold was designed to create four bile ducts, each measuring 140 mm in length, with diameters ranging from 7 mm to 10 mm and a wall thickness of 1-1.5 mm. Commercially available metal rods with a 6 mm diameter were inserted into the parallel holes of the mold before pouring the silicone [13]. To prevent sticking, the mold and the metal rods were initially sprayed with Ease Release 200 (Sculpture Supply Canada, Toronto, ON). After waiting for ten minutes, Ecoflex™ 00-20 FAST silicone was poured into the mold (see Figure 4). The mold halves were clamped, and after 75 minutes, when the silicone cured, the vessels were removed from the rods. Two of these vessels were threaded through pairs of parallel holes in the 3D-printed mold, and syringes were used to fill the vessels with water before testing. 

**Figure 5:**
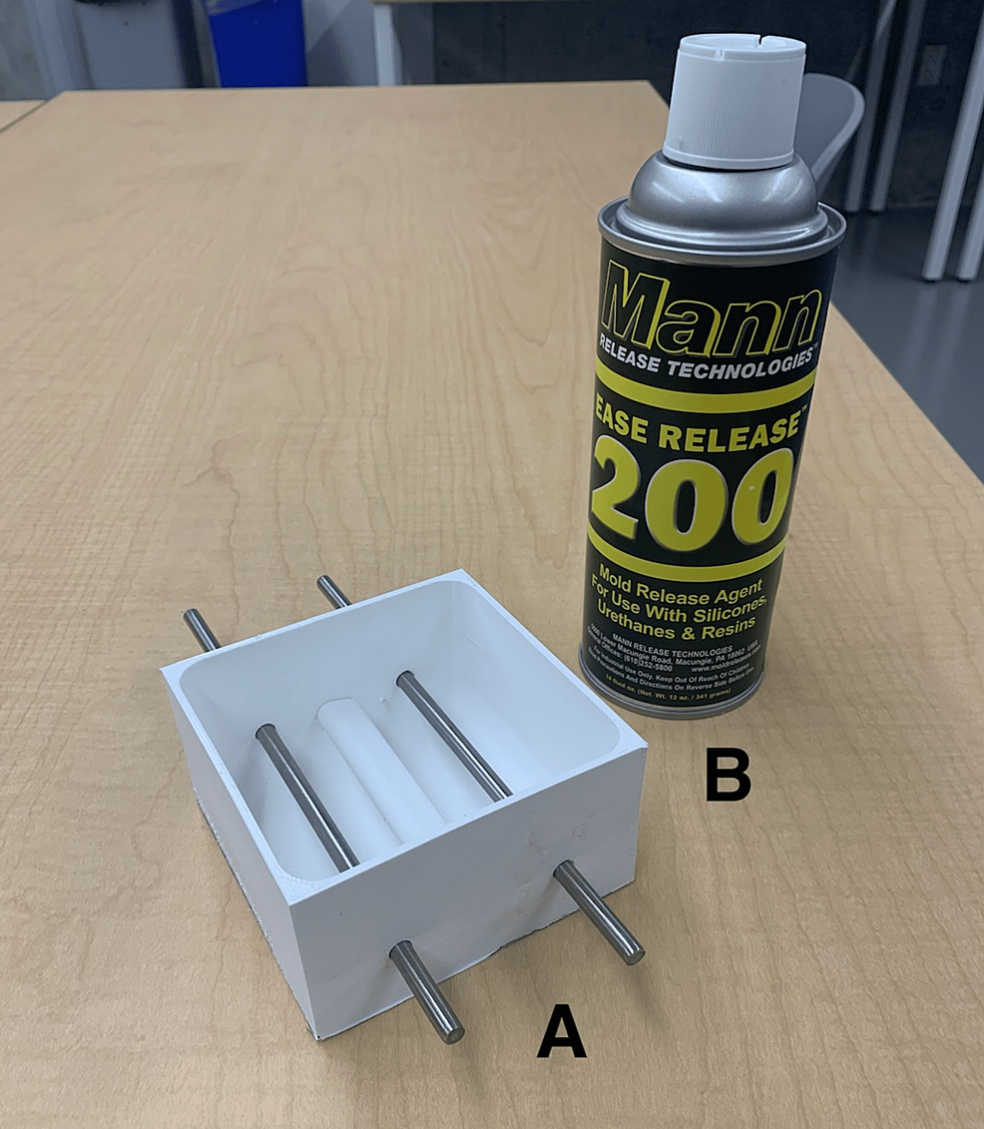
Materials used to create vessel type 3: A) metal rods inserted through the parallel holes in the 3D-printed mold, and B) Ease Release 200 used to spray the metal rods.

Vessel type 3: The third vessel type was a hollow canal through the silicone simulator, created using the same metal rods (6 mm diameter) as used for vessel type 2. All sides of the rods were sprayed with Ease Release 200. After ten minutes, and before pouring the degassed silicone, the rods were inserted through the parallel holes in the 3D-printed mold (see Figure 5). Once the silicone cured, the metal rods were withdrawn from the prototype, leaving a hollow canal with a singular wall. Plugs with a 7 mm diameter were designed using Fusion360™ and printed using Ecotough™ PLA filament material and the Ultimaker S5 3D printer. The hollow space was filled with water using syringes, and the vessels were sealed on both ends before testing.

Nerves: Two types of nerves were produced for testing. 

Nerve type 1: A single nerve strand was created using a single (1 mm diameter) Ecotough™ PLA filament. The PLA spool was loaded onto the Ultimaker S5 3D printer, and the material was allowed to extrude to create the filament. The filament was then cut into 20 cm segments, and one segment was placed in the 3D-printed mold before pouring the degassed silicone to form the nerve.

**Figure 6:**
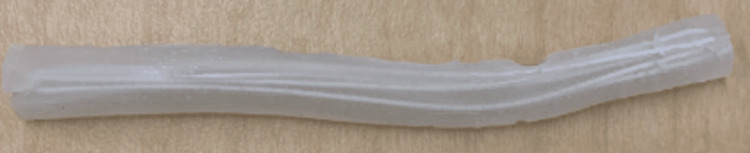
An example of a nerve bundle crafted using eight (1 mm diameter, 20 cm length) Ecotough™ PLA filaments, which were placed in the mold and covered in silicone. PLA, polylactic acid

Nerve type 2: A nerve bundle was crafted using eight (1 mm diameter, 20 cm length) Ecotough™ PLA filaments. The bundle was placed in the same mold used to create vessel type 2 (see Figure 6). Ecoflex™ 00-35 FAST silicone (Smooth-On, Macungie, PA, USA) was poured into the hollow space in the mold, and the eight filaments were held tightly together within the silicone for five minutes until it cured. After curing, the nerve bundle was removed from the mold, and the edges were evenly trimmed using a knife (see Figure 6). The nerve bundle was then threaded through a pair of parallel holes in the 3D-printed mold, and the degassed silicone was poured to create the simulator.

Two simulator prototypes were developed for subsequent testing, both made using degassed silicone, as described in Round 1. These prototypes are detailed in Table 3.

**Table 3:** Prototypes A and B created in Round 2. For both prototypes, this table demonstrates the vessel type (column 2) and nerve type (column 3) used in the design.

Prototype	Vessel type	Nerve type
A	Type 1, Type 3	Type 2
B	Type 2, Type 3	Type 1

**Figure 7:**
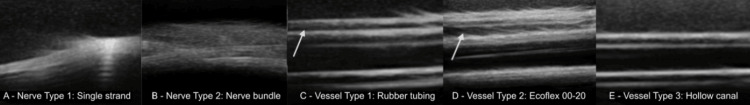
Single strand nerve type 1 (panel A), nerve bundle type 2 (panel B), rubber tubing vessel type 1 (panel C), Ecoflex 00-20 type 2 (panel D), and hollow canal vessel Type 3 (panel E) The images of nerve types 1 and 2 in panels A and B support the expert’s judgment that type 2 (panel B) was considered to be more realistic than the single PLA strand type 1 (panel A). The images of vessel types 1, 2, and 3 in panels C, D, and E support the expert’s judgment that type 3 (panel E) was considered to be the most realistic as there was no double wall when this vessel was imaged. Vessel types 1 (panel C) and 2 (panel D) led to an image that showed a double wall indicated by the white arrows in each panel. PLA, polylactic acid

Results: The results of testing prototypes A and B revealed that the nerve bundle (type 2) was considered to be more realistic than the single PLA strand (type 1) (Figure 7). In addition, vessel types 1 and 2 led to an image that showed a double wall (Figure 7). This was not observed when vessel type 3 was imaged, which was considered the optimal vessel type (Figure 7). Although the image quality was best for type 3, the clinical applications specialist suggested that when types 1 and 2 were used for simulated cannulation, the haptic feedback from the needle penetrating the walls of the simulated vessels provided a more realistic feeling than when vessel type 3 was cannulated. 

Round 3

The central aim of the third round was to evaluate how various 3D printing materials and architectural factors, such as shape and casing, influenced the image quality when bones were integrated into the silicone.

Methods: When using ultrasound to image a rigid object, there can be minute vibrations caused by the interaction of high-frequency sound waves with the object. While these vibrations are typically imperceptible, the resulting echoes may distort image quality. Bone rigidity can significantly impact image quality. Hence, in Round 3, we manipulated bone rigidity by altering the shape of the bones, the materials used, and whether the bones were enclosed within a special casing. To achieve this, the research team crafted twelve prototypes using silicone.

Materials: Two materials were employed: Ecotough™ PLA filament material and photopolymer resin. The PLA bones and those fused with the casing were designed with TinkerCAD™ (Autodesk Inc., San Rafael, CA, USA) and produced using an Ultimaker S5 3D printer. The bones created with Elegoo Standard Grey Resin were designed using SolidWorks 2022, featuring a layer thickness of 0.050 mm and an exposure time of 8 seconds per layer. These prints were subjected to an isopropyl alcohol wash and exposed to UV light for 20 minutes.

**Figure 8:**
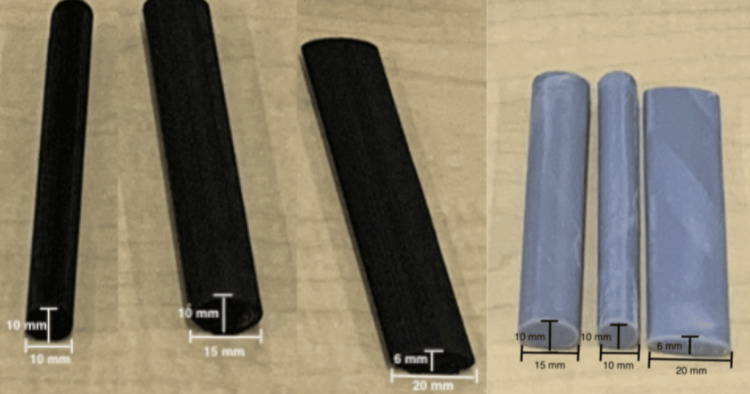
This figure illustrates the cross-sectional dimensions of the three types of PLA bones (in black), and the three types of resin bones (in grey) tested in Round 3. PLA, polylactic acid

Shapes: Three different bone shapes (round, oval, flat) were printed with each of the two printing materials. Each bone measured 95 mm in length, with cross-sectional dimensions illustrated in Figure 8. 

**Figure 9:**
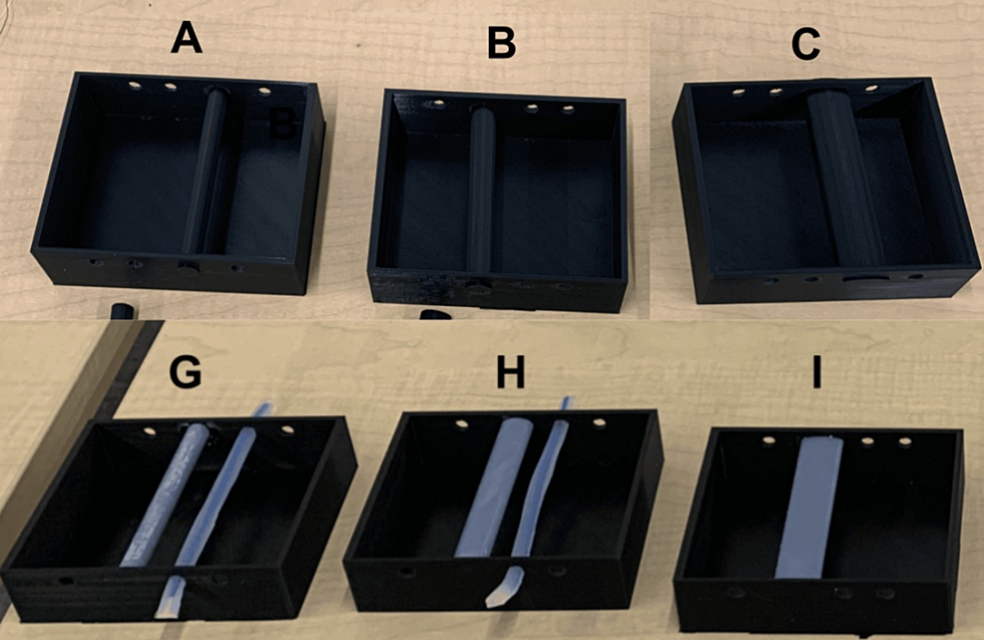
The top row (A, B, and C) shows how the three PLA bones (A - round, B - oval, and C - flat) were digitally fused with PLA casing to increase the overall stiffness of the system to minimize acoustic resonance. The bottom row (G, H, and I) shows how the three resin bones (G - round, H - oval, and I - flat) were glued to PLA casing to increase the overall stiffness of the system to minimize acoustic resonance. PLA, polylactic acid

Casing: The casing was produced using Ecotough™ PLA filament material, with identical dimensions to the mold used in Rounds 1 and 2. Before printing, we digitally integrated the design of the mold and the three bone shapes made with PLA (see Figure 9). The bones printed with resin material were affixed into the casing using superbond adhesive (see Figure 9).

In summary, 12 bone prototypes were created in total, each placed within the silicone to generate the 12 prototypes described in Table 4. All prototypes were produced using degassed silicone, as detailed in Round 1. Please refer to Table 4 for additional information.

**Table 4:** Experimental conditions for testing the 12 prototypes created in Round 3, resulting from the combinations of shape, materials used, and inclusion of casing in the design of the bones. Column 1 indicates the prototype, column 2 indicates the shape of the bone, column 3 indicates the material used for the construction of the bone, and column 4 indicates whether the bone was fused with the casing or not. PLA, polylactic acid

Prototype	Shape	Material	Casing
A	Round	PLA	Yes
B	Oval	PLA	Yes
C	Flat	PLA	Yes
D	Round	PLA	No
E	Oval	PLA	No
F	Flat	PLA	No
G	Round	Resin	Yes
H	Oval	Resin	Yes
I	Flat	Resin	Yes
J	Round	Resin	No
K	Oval	Resin	No
L	Flat	Resin	No

**Figure 10:**
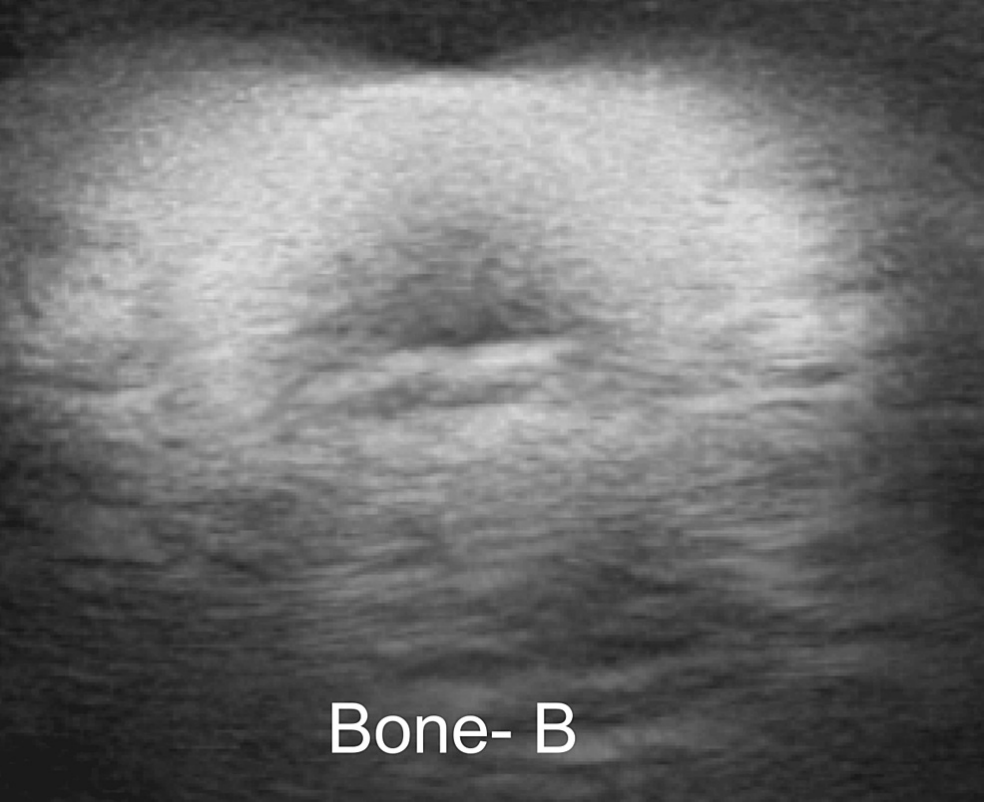
This figure illustrates the quality of the image generated from prototype B, which was constructed using an oval-shaped PLA bone digitally fused with the casing. PLA, polylactic acid

Results: The results of testing showed that prototype B, the oval-shaped PLA bone printed with the casing provided the most clear and realistic image (Figure 10). 

**Figure 11:**
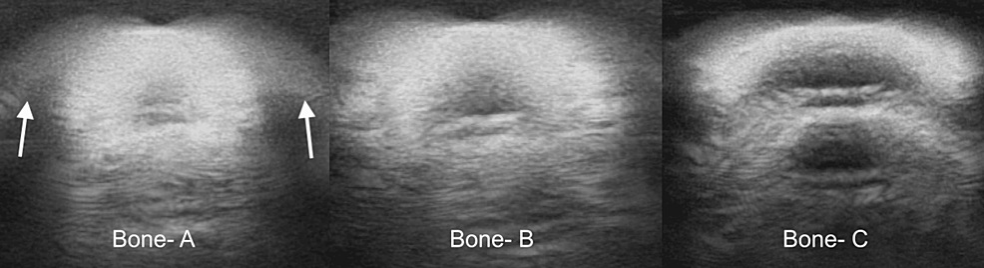
Prototypes A (left), B (middle), and C (right). This figure demonstrates the distortions and shadows created when the round-shaped PLA bone was imaged, which were attributed to the process of 3D printing. White arrows show the distortions and shadows created by the round-shaped bone. PLA, polylactic acid

A comparison of prototypes A, B, and C showed that the circle-shaped PLA bones created distortions and shadows that were attributed to the process of 3D printing (Figure 11). Specifically, the 3D printers print in lined layers, which creates small grooves when printing the curvature of the bones, known as the staircase effect [14].

**Figure 12:**
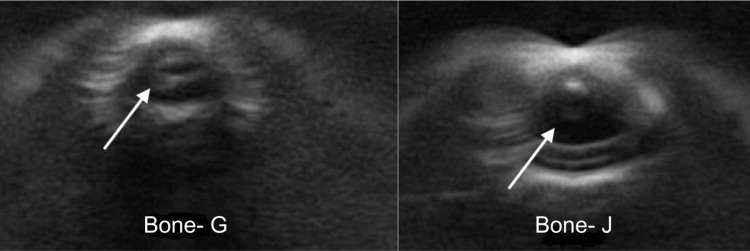
Comparison of prototype G (with casing) on the left and prototype J (without casing) on the right. White arrows show the resin bones presenting as a hollow space. Gluing the bones into the casing, as demonstrated by prototype G (left), did not minimize this effect.

The resin bones presented as a hollow space under ultrasound imaging, which was similar to that of a vessel. A comparison of the resin prototypes with and without casing showed that gluing the bones into the casing did not minimize this effect (Figure 12). 

**Figure 13:**
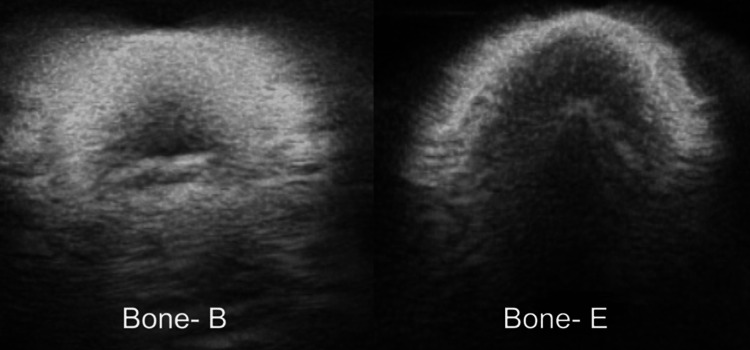
Comparison of the oval bones in prototype B (with casing) on the left, and prototype E (without casing) on the right. This figure demonstrates the reduced distortions in prototype B, where the bone was digitally fused with the casing.

Bones integrated within the casing exhibited the highest degree of rigidity, showcasing minimal shadows and distortions during imaging. A comparison between the PLA prototypes, both with and without the casing, revealed a significant reduction in vibrations and distortions due to the casing, leading to improved image quality (see Figure 13). These observations align with the findings of Jegennathan et al., who noted the presence of acoustic shadows in ultrasound imaging when silicone phantoms containing 3D-printed bones were subjected to ultrasound [15].

One plausible explanation for this observation is that ultrasound waves induce resonance frequencies in the bones, resulting in the appearance of shadows. In the absence of constraints, the bones have the freedom to move within the silicone when subjected to sound waves. That is, it is conceivable that ultrasound waves initiate vibrations in the bones, causing them to shift in position. These vibrations, in turn, contribute to distortions and shadows within the image. This resonance effect can significantly degrade image quality. 

Outcome

The comprehensive findings from Rounds 1, 2, and 3 indicate that the silicone-based simulator with the following components is the most suitable for more anatomically correct designs and further assessments of functionality:

Vessels

The hollow canal (type 3) emerged as the optimal choice for superior ultrasound visualization and cost-effectiveness. The rods utilized for crafting these hollow vessels were priced at $16.90 CAD (taxes included). Importantly, since these rods are reusable, they can be employed to create multiple simulators.

Nerves

The nerve bundle (type 2) exhibited the most lifelike imagery and proved to be a standout feature in the simulator's design. The cost associated with the nerves amounted to approximately $0.55 CAD (taxes included).

Bones

The oval-shaped bone enclosed in a casing provided the best visualization while effectively minimizing vibrations and distortions. The combined cost of the bone and casing was $5.56 CAD (taxes included), with the bone alone costing $0.60 CAD (taxes included).

Hence, the final prototype should involve silicone degassing employing a single-stage process, featuring hollow canal vessels, nerve bundles, and oval bones enclosed within a casing. The total cost of this simulator, exclusive of research, but including labor and any other direct expenses incurred during fabrication (with taxes), stands at $18.11 CAD.

## Discussion

This technical report outlined the development of an affordable ultrasound simulator designed to support the TTT model for ultrasound training. The simulator was crafted using readily available materials following additive manufacturing techniques and adhered to the design-to-cost approach, where the team was focused on optimizing cost-efficiency without sacrificing accessibility, making it a cost-effective alternative to the CAE Blue Phantom [16]. Collaborating closely with FUJIFILM Sonosite Canada Inc., there were numerous design iterations, and each iteration underwent evaluation by a clinical applications specialist, ultimately yielding an acceptable final product, as deemed by the clinical applications specialist. 

Body of the simulator

The most significant and unique contribution of this work in the area of construction of the body of the simulator is in the degassing process. First, we have shown that second stage degassing, once the degassed silicone was placed in the 3D-printed mold and degassed again, resulted in entrapment of air bubbles. We speculate that this is because air particles were trapped in the 3D printing process, despite 100% infill setting, and in negative pressure (i.e., vacuum) these particles escaped the mold into the silicone. Therefore, we recommend that the degassing process should be a single-stage process before pouring the silicone into the mold. When using ballistic gel, the degassing process did not result in any significant changes in the amount of air particles trapped, and therefore degassing may not be necessary.

Vessels

**Figure 14:**
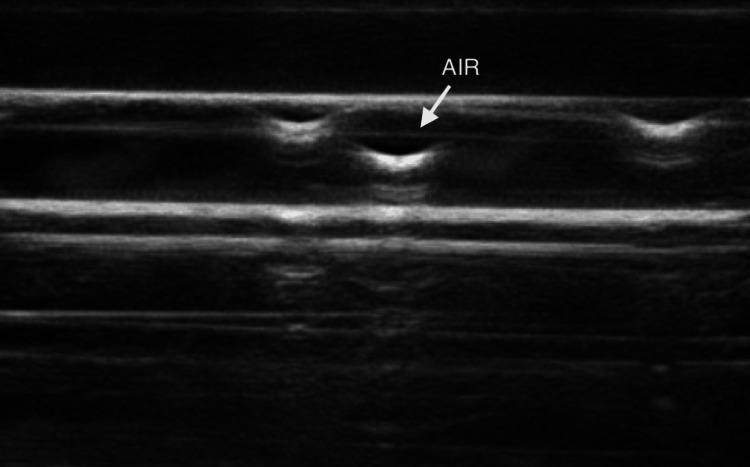
Air pockets shown in the hollow canal vessels. The white arrow shows an air pocket. This figure shows the air pockets created by trapped silicone remnants, which were caused by removing the metal rods from the silicone prototype.

One notable area for future development and focus is the creation of vessels. Past ultrasound simulators often featured wall-less or hollow canal vessels [17]. However, when a technique was developed and applied to produce similar wall-less canal vessels here, we encountered a drawback whereby the removal of metal rods after the curing of silicone occasionally left trapped silicone remnants, resulting in visible air pockets in ultrasound images (see Figure 14). Additionally, the hollow canal design lacked haptic feedback, although it aligns with the "gold standard" represented by the CAE Blue Phantom. The acceptability of the absence of haptic feedback in training will be assessed in further testing, guided by the design-to-cost approach [16].

Nerves

The nerve bundle within the simulator emerged as a notable innovation. While nerves have previously been produced as single strands, our team pioneered the construction of nerve bundles using multiple PLA filaments that were casted in silicone [18]. To increase the contrast between the nerve bundles and the rest of the silicone body, silicones of different durometry (hardness or softness) can be used. To create silicone with different levels of durometry, one can use a two-component silicone rubber system, which typically consists of a base component and a curing or catalyst component. By adjusting the ratio of these components and sometimes using additives, you can achieve the desired durometery value.

In this technical report, the general process for making silicone with different durometry involved a two-component silicone system (Smooth-On, Macungie, PA). First, in order to obtain a baseline duromentry, we measured the desired amounts of both parts according to the manufacturer's recommendations. Next, we used a durometer tester (e.g., Boaby, Digital Shore Hardness Tester Digital Durometer Scale for Silicone Tire Rubber Foam Type A Hardness Tester 0-100 HD Resolution: 0.1HA) to measure the durometer value of the baseline silicone. Adjusting the durometer value of subsequent silicone samples may be achieved by altering the ratio of the two parts of the silicone system. To make the silicone softer, one needs to add more catalyst parts (typically part B); to make it harder, add more base parts (typically part A). The specific mixing ratio will depend on the silicone product and the desired durometer value.

Bones

The introduction of a casing to hold the bones was found to enhance the quality of ultrasound images. This improvement is attributed to the hypothesis that the initial image noise was linked to vibrations in the free-floating printed bones. By affixing the bones to a sturdy casing, these vibrations were mitigated. Conversely, the shift from fused filament PLA to photopolymer resin did not yield positive effects and instead appeared hollow in images, which warrants further investigation. Historically, ultrasound simulator bones have been crafted from various materials, including resin, nylon, gypsum, and PLA [19]. We are among the first to experiment with different bone shapes embedded in the phantom and the inclusion of a casing.

Future directions

Subsequent developments will encompass the creation of an anatomically accurate simulator, exploration of alternative materials and printing parameters for rigid structures, and an in-depth analysis of materials at varying depths within the silicone [6]. The next steps include integrating the maxSIMghost simulator into an ultrasound curriculum to assess its clinical educational value and acceptability.

Once finalized, the current simulator and its components will be made available on our laboratory’s GitHub repository of designs (https://github.com/maxsimhealth/). These designs are published with a CC license (Attribution-NonCommercial-ShareAlike 4.0 International, https://github.com/maxSIMhealth/OntarioTechUNur101/blob/main/LICENSE.md), which permits their use for educational and research purposes. The license strictly prohibits all commercialization of our designs in their current and modified forms. Currently, we are also developing a partnership model for knowledge and technology mobilization for training sites that may not have access to equipment used to develop the simulator [20]. Currently, there is no such partnership model for transferring simulation tech, such as the one described in this technical report, from universities to healthcare settings. The proposed solution advocates for a partnership model involving university research centers, for-profit and not-for-profit sectors to facilitate the integration of simulation technology into the health professions training sector. This model should circumvent small enterprises from using the designs to generate profit by selling such simulators to training sites.

## Conclusions

This technical report underscores the feasibility of designing and constructing cost-effective, standardized simulators that produce realistic ultrasound images suitable for training. Our work represents a pioneering effort in systematically testing materials, vessels, nerves, and bones in a single simulator while adhering to the design-to-cost approach. Over three rounds of testing, we have ascertained that hollow canal vessels, PLA nerve bundles, and PLA bones enclosed in a casing enhance the quality of ultrasound-generated images while optimizing cost-efficiency and offering design flexibility. However, further research is essential to explore the implications of these design characteristics when anatomical components are embedded in anatomically accurate simulators (e.g., arm or knee), including the impact of shadows and their depth.
